# Hypothyroidism Induced Severe Rhabdomyolysis in a Hemodialysis Patient

**DOI:** 10.1155/2014/501890

**Published:** 2014-04-07

**Authors:** Erhan Tatar, Tolgay Isikyakar, Kezban Pinar Yeniay, Hasan Huseyin Uzuner, Ebru Sevinc Ok

**Affiliations:** ^1^Division of Nephrology, Izmir Bozyaka Education and Research Hospital, 9035170 Izmir, Turkey; ^2^Department of Internal Medicine, Izmir Bozyaka Education and Research Hospital, 9035170 Izmir, Turkey

## Abstract

Hypothyroidism occurs relatively common and is a significant cause of morbidity and mortality during the course of chronic kidney disease. Rhabdomyolysis is a potentially life-threatening condition characterised by necrosis of muscular tissue and rarely associates with hypothyroidism. Here we describe a case of rhabdomyolysis due to severe hypothyroidism in a 56-year-old female hemodialysis patient.

## 1. Introduction

Thyroid dysfunction is relatively common in patients with chronic kidney disease (CKD) when compared to general population [1, 2]. Both hormonal changes including alterations in TRH, TSH, and iodine clearance as well as presence of associating autoimmune disorders (type 1 diabetes mellitus or systemic lupus erythematosus) and comorbidities such as HCV infection or treatment with drugs having adverse thyroid effects (e.g., amiodarone) are thought to be responsible for thyroid dysfunction [2–4]. Thyroid dysfunction particularly hypothyroidism is a significant cause of cardiovascular mortality and morbidity in CKD patients [5–10]. In hemodialysis patients, however, the frequency of acute complications and neuromuscular effects of hypothyroidism are not known.

Rhabdomyolysis is a rapid breakdown of skeletal muscle tissue leading to release of its contents into systemic circulation [11]. Rhabdomyolysis, a life-threatening condition, may occur due to physical factors including trauma, convulsions, or overexertion as well as to chemical and hormonal causes [11]. Hypothyroidism associated rhabdomyolysis is rare in nonuremic patients. Hypothyroidism induced rhabdomyolysis in dialysis patients has not been reported as far as we know. Here we present a case of rhabdomyolysis in a hemodialysis patient on amiodarone treatment receiving antithyroid therapy for subclinical hyperthyroidism.

## 2. Case Presentation

A 56-year-old female with a past medical history of end stage diabetic nephropathy, interstitial pulmonary disease, congestive heart failure, and atrial fibrillation presented to nephrology outpatient clinics with complaints of nausea and fatigue. She was back on routine hemodialysis 4 times a week for 18 months (she underwent a renal transplantation 10 years ago). She provided a history of subclinical hyperthyroidism detected six months ago for which antithyroid treatment was started because of the diagnosis of a thyroid nodule. She stated that she missed her follow-up appointments. Her medications included warfarin 5 mg, diltiazem 30 mg, amiodarone 400 mg (started for atrial fibrillation with rapid ventricular response), propylthiouracil 300 mg, theophylline 300 mg, acetylsalicylic acid 100 mg, olanzapine 2.5 mg, citalopram 10 mg, and calcium acetate 1500 mg daily.

**Figure 1:**
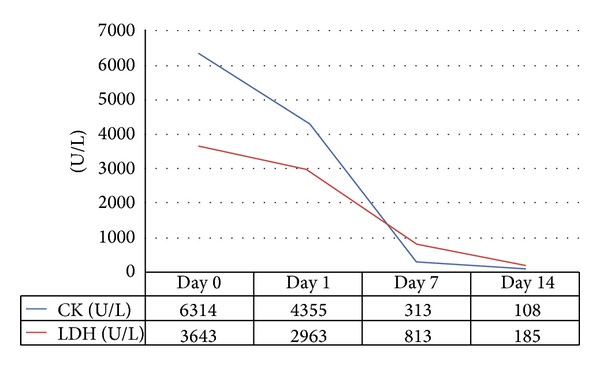
Rhabdomyolysis parameters of the patient during the hospital stay.

On admission her blood pressure was 80/50 mmHg, body temperature was 36.7°C, pulse rate was 50 beats/min, and respiratory rate was 12/min. Physical examination revealed bilateral rales at the lung bases. Electrocardiogram showed atrial fibrillation with slow ventricular response with 45–50 bpm. Upon initial assessment laboratory studies revealed the following: hemoglobin: 10.8 g/dL, mean corpuscular volume (MCV): 78, total leukocyte count: 13,700/mm3, and platelet count: 289 × 10^9^/L; random blood sugar: 117 mg/dL, blood urea: 142 mg/dL, serum creatinine: 6.61 mg/dL, serum albumin: 4.06 g/dL, serum aspartate amino  transferase (AST): 400 (0–31) U/L, serum alanine amino transferase (ALT): 71 (0–31) U/L, creatine phosphokinase (CPK): 6314 (26–192) U/L, CPK-MB: 12 (2–24) U/L, troponin I: 0.252 ng/mL (0–0.06), serum lactate dehydrogenase (LDH): 3643 (135–214) U/L, serum sodium: 132 mEq/L, and serum potasium: 7.2 mEq/L; TSH: >100 uIU, free T3: 1.5 pg/mL, and free T4: 0.4 (repeated twice). Antithyroglobulin and antithyroid peroxidase antibody were negative. Basal serum cortisol level was 32.97 ug/dL (6.7–22.6). Thyroid gland size was measured to be normal on thyroid ultrasound while a heterogeneous appearance was observed including fibrous bands with no nodules. She was diagnosed with hypothyroidism induced rhabdomyolysis. Thyroid function test results during hemodialysis treatment are demonstrated in Table 1. Antithyroid and antiarrhythmic medications were discontinued. Levothyroxine replacement therapy was started with a daily dose of 100 mcg, which then gradually increased up to 200 mcg. The patient underwent daily hemodialysis during the first five days of the hospital stay. On hospital followup the patient's muscle enzymes gradually declined to normal ranges in nearly two weeks (Figure 1). The patient was discharged in good clinical condition after two weeks of hospitalization.

## 3. Discussion

Hypothyroidism related skeletal muscle involvement is observed in almost 80% of the nonuremic patient population in which muscle serum creatinine kinase is usually slightly elevated [12]. Deterioration of glycogenolysis, mitochondrial oxidative metabolism, and triglyceride turnover in thyroxine deficiency may be the responsible pathogenetic mechanisms. Hypothyroidism associated rhabdomyolysis, on the other hand, is quite rare [13]. Hypothyroidism induced rhabdomyolysis, depending on its severity, may be complicated with acute kidney injury [14]. Rhabdomyolysis tends to occur more frequently in a portion of comorbid patients particularly receiving antihyperlipidemics (i.e., statins and fibrates) [15, 16]. In the literature, however, there have been no reports regarding hypothyroidism induced rhabdomyolysis in a hemodialysis patient. Hemodialysis patients are likely to have an increased risk for hypothyroidism induced rhabdomyolysis regarding the presence of comorbidities such as electrolyte imbalances, diabetes mellitus, medications received, and drug-drug interactions (i.e., antihyperlipidemics, antihypertensives, and antiarrhythmics).

Kidneys play a significant role in thyroid hormone metabolism. Deterioration in kidney functions may lead to important alterations in thyroid functions for which a variety of mechanisms have been suggested [3, 4]. The most common thyroid function abnormality in patients with CKD is euthyroid sick syndrome (i.e., low T3 syndrome) and hypothyroidism frequency of which increases with the severity of CKD [1, 2]. The frequency of subclinical hyperthyroidism, on the other hand, is the same as the general population [3, 17]. However, there is not enough information about whether to start antithyroid treatment in CKD patients with subclinical hyperthyroidism. The decision to start antithyroid treatment should be given carefully considering that thyroid function parameters in case of comorbid situations tend to vary during the course of followup, and that these medications are likely to interact with others in CKD patients, who usually are on multiple drugs. In our patient, hypothyroidism is likely to have developed due to antithyroid treatment started for subclinical hyperthyroidism.

Amiodarone, a class III antiarrhythmic drug, potentially interferes with normal thyroid function owing to its high iodine content [18, 19]. It inhibits both peripheral conversion of T3 to T4 via blocking 1–5′ deiodinase and the entry of thyroid hormone into peripheral tissues, thereby increasing serum fT4 levels while lowering serum fT3 levels. Amiodarone alters pituitary synthesis and release of TSH and inhibits the activity of 2–5′ deiodinase resulting in elevated serum TSH concentrations [18, 20]. The majority of the patients treated with amiodarone remain euthyroid whereas the treatment sometimes may lead to thyrotoxic or hypothyroid states. Amiodarone induced clinical hypothyroidism is seen in only 5% of the patients receiving the treatment. The predisposing factors are coexisting Hashimoto's thyroiditis and/or presence of autoantibodies as well as living in iodine-sufficient areas [19]. Long-term amiodarone treatment is not likely to be solely responsible for the severe hypothyroidism in our patient. The antithyroid medication, kidney disease, and decreased clearance of iodine are possible contributors. Amiodarone, which is metabolized by cytochrome P-450 (CYP) 3A4 in the liver, may interact with concurrent drugs (i.e., antihyperlipidemics) by interfering with their metabolism and this may lead to adverse events including rhabdomyolysis [21]. Our patient, although denied a medical history of use of antihyperlipidemic drugs, was on multidrugs, and we think that potential drug interactions could have contributed to development of rhabdomyolysis.

In conclusion, thyroid dysfunction is an important problem in hemodialysis patients. Hypothyroidism induced rhabdomyolysis may be seen, although rarely, in these patients. Hypothyroidism must be questioned in the etiological assessment of rhabdomyolysis in hemodialysis patients.

## Figures and Tables

**Table 1:** Thyroid function test results during hemodialysis treatment.

	Eighteen months prior to admission	Six months prior to admission (on propylthiouracil treatment)	Upon admission	One month after admission (on levothyroxine treatment)
TSH (normal range) (0.41–4.25 uIU/mL)	0.80	0.04↓	>100↑↑	2.44
fT3 (normal range) (2.5–3.9 pg/mL)	2.40↓	2.30↓	1.5↓↓	2.09↓
fT4 (normal range) (0.61–1.06 ng/dL)	1.05	0.98	0.4↓↓	1.53↑
Antithyroglobulin (0–100 IU/mL)		neg	0 (neg)	
Anti-TPO (0–100 IU/mL)		neg	0.9 (neg)	
